# Study on valuable metal incorporation in the Fe–Al precipitate during neutralization of LIB leach solution

**DOI:** 10.1038/s41598-021-02019-2

**Published:** 2021-12-02

**Authors:** Alexander Chernyaev, Benjamin P. Wilson, Mari Lundström

**Affiliations:** Department of Chemical and Metallurgical Engineering, School of Chemical Engineering, Aalto University, 00076 Aalto, Finland

**Keywords:** Chemistry, Chemical engineering

## Abstract

The role of aluminum concentration and pH in the purification of waste Li-ion battery leach solution was investigated using NaOH and LiOH as neutralization agents (*[H*_2_*SO*_4_*]* = 0.313 M, *t* = 6 h). Solution was prepared from synthetic chemicals to mimic real battery leach solution. Results demonstrate that pH (3.5–5.5) has a significant effect on the precipitation of metals (Fe, Al, Ni, Cu, Co, Mn, and Li), whereas higher temperature (*T* = 30 and 60 °C) decreases the precipitation pH of metals. Iron and aluminum were both found to precipitate at ca. pH 4 and the presence of aluminum in PLS clearly decreased the separation efficiency of Fe vs. active material metals (Ni, Co, Li). In the absence of dissolved aluminum, Fe precipitated already at pH 3.5 and did not result in the co-precipitation of other metals. Additionally, the Al-free slurry had a superior filtration performance. However, aluminum concentrations of 2 and 4 g/L were found to cause loss of Ni (2–10%), Co (1–2%) and Li (2–10%) to the Fe-Al hydroxide cake at pH 4. The use of LiOH (vs. NaOH) resulted in 50% lower co-precipitation of Ni, Co and Li. Overall, these results demonstrate that hydroxide precipitation can be an effective method to remove iron from battery waste leach solutions at aluminum concentrations of < 2 g/L only. Although the highest level of lithium loss in the cake was found at pH 4, the loss was shown to decrease with increasing pH.

## Introduction

Currently, the world is facing a major challenge because of anthropogenic climate change that demands significant steps towards less carbon-intensive and more sustainable lifestyles to be taken. One major strategy to achieve these goals is the increased electrification of transportation that is leading to the increased use of Li-ion batteries and the depletion of associated resources. For example, as of 2015, in Europe vehicles accounted for around 45% of all CO_2_ emissions^1^, which has led to an increased legislative focus on accelerating the transition to electric vehicles (EVs) and hybrid electric vehicles (HEV) that require Li-ion batteries (LIBs)^2^. Additionally, LIBs have great potential to serve as energy storage to support microgrids in producing electricity from renewable sources (solar and wind energy)^3–5^. Consequently, this growth in EVs and energy storage means many LIBs will need to be produced to meet future demand.

A LIB cell comprises active material (Co, Ni, Mn, Li) coated on aluminum current collector, serving as a cathode, whereas the anode current collector typically comprises copper foil coated with a graphite/lithium mixture^6^. Recent research on battery recycling has focused on the recovery of critical elements like Li, Co, Mn, Ni and C (graphite) via several stages that include pretreatment, such as dismantling, crushing, screening and potentially thermal treatment to remove organic binders^7^. Current industrial scale recycling methods typically involve preliminary dismantling to remove the outer casing (metallic and plastic) to make the cells available for further processing in the case of large batteries, such as EV or power tools, whereas small batteries can be processed without preliminary dismantling^7–14^. Cells are crushed and sieved in the initial treatment stages as this removes the need for complicated battery cell opening and therefore, all batteries in the incoming stream can be readily pre-treated in large volumes. The mechanical and thermal pretreatment enables partial removal of current collectors and toxic volatile components, such as LiPF_6_ electrolyte, organic solvents, and binder (PVDF)^15,16^. The metal-rich black mass is subjected to further treatment by mechanical separation, pyrometallurgical and/or hydrometallurgical processes^8,17,18^.

Nevertheless, shredding of battery pack with minimum prior dismantling produces very complex black mass of low purity—composed of a mixture of the active material, Fe, F^−^, Al, Cu, plastics, and graphite^19^—that is not necessarily conducive to the subsequent materials recovery. The presence of Al, Cu and Fe fragments can benefit leaching processes as they serve as efficient reducing agents for the active material, and thus the use of externally introduced reducing agents, like hydrogen peroxide, can be omitted^19,20^. Of these elements, Al is the most challenging to remove from the solution, although the purity of the recovered elements, especially lithium and graphite, must be of pristine quality which can be a challenge to achieve in the presence of such metal fragment impurities^8,16,21–25^. Moreover, the active material as well as other elements in leaching can be a major source of impurities for recovered graphite, such that the reuse in battery applications is precluded^26^. Consequently, an industrial-scale disassembly and battery pack layer delamination technology can potentially reduce the processing costs as all layers can be separated prior to metallurgical processing aimed at battery grade purity. Such technologies could allow for sustainable recycling or reuse of battery components^15^.

Although combined pyrometallurgical-hydrometallurgical processes have been widely investigated—and used—the downside of pyrometallurgical approaches is the loss of lithium, manganese, carbon, electrolyte and polymers in the slag or energy reuse^15^. In contrast, hydrometallurgical acidic leaching processes result in pregnant leach solutions (PLS) rich in metals^7,27^ as in addition to the critical battery metals (Co, Ni, Mn, Li) also electrode materials and wiring (Cu, Al) as well as impurities (e.g., Fe originating from the casing and F^−^) all dissolve into the solution^17,19,28–31^, while graphite and plastic fragments remain in the leach residue. Due to such complexity and low purity of black mass, leach solutions need to undergo intensive, multi-step purification through neutralization and purification, solvent extraction, and crystallization methods^27^ to recover the desired elements. Typically, sodium hydroxide is used for both neutralization and iron and aluminum removal directly after the leaching^29,32,33^. At this stage of the process, selectivity is a critical factor, as overall process performance is highly dependent on any losses of valuable battery metal (Co, Ni, Li) materials into the Fe-Al precipitates.

Conventionally, both iron and aluminum are considered impurities in hydrometallurgical processing and these metals are seldom recovered as products, but rather end up in the waste residues. Peng et al.^28^ have previously reported that aluminum foil in the overflow produced by sieving LIB scrap can comprise up to 11 wt% of cobalt, therefore the discarding aluminum current collector scrap can also inadvertently result in cobalt loss. Additionally, aluminum content in commercial LIBs ranges from 3 to 22 elemental wt% and cannot be completely removed by sieving—even when sieved, black mass can contain up to 12 wt% of aluminum^20^. After battery scrap crushing fine Al particles end up in the underflow, whereas the coarser aluminum foil fragments—often with entrained LiCoO_2_ and LiNi_x_Co_y_Mn_z_O_2_—are found in the overflow^22,28,34^. Moreover, LiNi_1-x-y_Co_x_Al_y_O_2_ (NCA) type active material is gaining popularity due to the effective substitution of Ni for Al, high theoretical capacity and lower cost^35,36^. Consequently, aluminum is inevitably present within the leaching process and the resultant PLS^19^.

Co-precipitation of valuable metals (Li, Ni, Co, Cu, and Mn) during iron and aluminum removal by hydroxide precipitation and filtration efficiency is known to occur^6,31,37,38^. Such behavior occurs as a result of the formation of aluminum hydroxide (Al(OH)_3_) precipitate, which may also absorb other metals into the cake (precipitate) as pH increases, which leads to decreased process selectivity^22,29,39^. Therefore, to gain a deeper understanding, the current work investigates the co-precipitation of valuable metals (Ni^2+^, Co^2+^, Cu^2+^, Mn^2+^, and Li^+^) as a function of PLS Al^3+^ concentration during Fe–Al-hydroxide precipitation induced by NaOH. Furthermore, to avoid the introduction of Na^+^ impurity into the system, LiOH was also investigated as an alternative precipitation agent.

## Materials and methods

### Reagents and synthetic pregnant leach solution

Synthetic PLS was prepared to mimic the real PLS from the leaching of LiCoO_2_-rich battery waste^17,19^, Table 1. The reagents used were Li_2_SO_4_∙H_2_O (Sigma Life Science, ≥ 99%), NiSO_4_∙6H_2_O (Alfa Aesar, 98%), MnSO_4∙_H_2_O (VWR chemicals, ACS/Reag. Ph.Eur.), CoSO_4_∙7H_2_O (Alfa Aesar, 98%), Al_2_(SO_4_)_3_∙14–18 H_2_O (Alfa Aesar, 97 + %), CuSO_4_∙5H_2_O (Sigma-Aldrich, ≥ 98%), Fe_2_(SO_4_)_3_∙nH_2_O (VWR chemicals, GPR Rectapur). Sulfuric acid (H_2_SO_4_, 95–97%, VWR Chemicals) was used to prepare the leach solution with a target concentration of 0.313 M H_2_SO_4_. Furthermore, it is assumed that copper removal was conducted prior to Fe–Al-hydroxide precipitation, therefore only traces of dissolved copper (ca. 100 ppm) was present in the initial synthetic PLS.

**Table 1:** Synthetic PLS composition (g/L).

Li^+^	Ni^2+^	Mn^2+^	Co^2+^	Al^3+^	Cu^2+^	Fe^3+^
6.7	3.6	3.1	47	0–4	0.1	1.3

### Precipitation test procedure

Precipitation tests were carried out in 200 mL conical flasks on a multi-stirrer (IKA RT 10). The initial solution volume was 85 mL, and it was pre-heated to either 30 °C or 60 °C and the agitation rate was kept constant at 620 rpm. Each conical flask corresponded to a pre-selected target pH value (3.5, 4, 4.5, 5, 5.5) in the range where Fe^3+^ and Al^3+^ can be fully precipitated^29,33^. The Al^3+^ concentration in the solution was variable (0, 2 and 4 g/L). The precipitating agent (2 M NaOH or LiOH) was added to the solution stepwise, and the pH was measured. Once the target pH was attained in each flask, the reaction time was initiated, and the experiments were continued for a total duration of 6 h. The pH in each flask was monitored hourly and adjustments to maintain the target pH value were undertaken using 4 M sulfuric acid as necessary.

Once the experiments were complete, a sample of known volume (4 mL) was siphoned from each flask while stirring and filtered through a 0.8 µm syringe filter. The resultant filtrate was immediately diluted in 0.2 M HNO_3_ to minimize the risk of unwanted precipitation. Once the liquid sample was extracted, a slurry sample was taken under agitation (15 mL) and subjected to filtration to obtain the cake. The final volume of the slurry was then measured and used in the following metal yield calculations. The filter paper used was Whatman grade 54 (22 μm) for Fe–Al-hydroxide precipitate separation and grade 50 (2.7 μm) in the case without aluminum. After the slurry filtration was complete, the cake was washed with acidified water with the corresponding target pH to avoid metal salt precipitation.

After rinsing, the individual filter cakes were dried in an oven for 10 h at 60 °C, before their total weight was measured and a sample of each cake (30–60 mg) was transferred to a separate 50 mL volumetric flask and dissolved in 0.5 M HCl at 80 °C. Once these solutions had been allowed to cool down to room temperature, they were analyzed with flame atomic absorption spectroscopy (AAS, Varian AA240). Prior to the AAS analysis, potassium (K^+^) ions were added to the standards, blank and samples to prevent Li^+^ ionization during the AAS determination. In addition, aluminum content was analyzed with an inductively coupled plasma optical emission spectrometer (ICP-OES, Perkin Elmer, Optima 7100 DV).

The total cake mass and related metal content (Ni, Co, Mn, Li) was calculated using Eqs. (1) and (2), whereas the precipitation of Al, Fe and Cu was calculated based on their content within the final solution via Eq. (3).

1 $$E\left(\%\right)=\frac{{m}_{cake}^{Me}\times {m}_{tot}^{cake}}{{c}_{i}^{Me}\times {V}_{i}^{sol}\times {m}_{sol}^{cake}\times 0.1},$$

where ${m}_{cake}^{Me}$ is the metal dissolved from the cake (mg), ${m}_{tot}^{cake}$ is the total cake weight (mg) calculated using Eq. (2), ${c}_{i}^{Me}$ is the initial metal concentration (g/L) in the leach solution, ${V}_{i}^{sol}$ is the volume (mL) of the starting leach solution, and ${m}_{sol}^{cake}$ is the mass of the cake dissolved in HCl.

The total cake amount was calculated based on the known amount of slurry taken (Eq. (2)).

2 $${m}_{tot}^{cake}=\frac{{m}_{s}^{cake}\times {V}_{f}^{sl}}{{V}_{s}^{sl}},$$

where ${m}_{s}^{cake}$ is the mass of the cake (mg) obtained by filtration, ${V}_{f}^{sl}$ is the final volume of the total slurry measured (mL), ${V}_{s}^{sl}$ is the volume of slurry taken for filtration (15 mL).

Precipitation of Fe, Al and Cu was calculated based on the metal weight in the initial and final solution using Eq. (3).

3 $$E\left(\%\right)=\frac{{(c}_{i}\times {V}_{i}\times 0.001)-({c}_{f}\times {V}_{f}\times 0.001)}{{(c}_{i}\times {V}_{i}\times 0.001)}.$$

## Results

**Figure 1:**
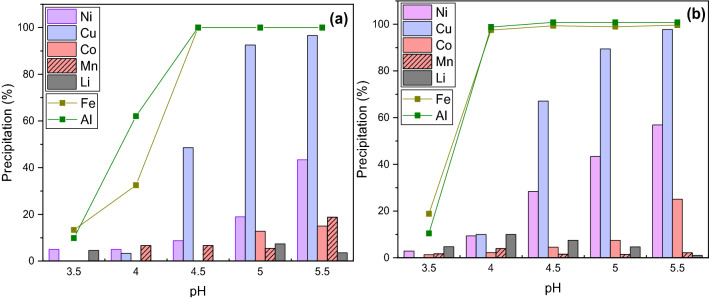
Precipitation using NaOH at (**a**)* 30 °C and (**b**) 60 °C with 4 g/L of Al in the solution (*based on solution analysis using Eq. (3)).

Synthetic LIB leach solution was used as the liquid raw material for precipitation research. During NaOH and LiOH addition, metals were shown to precipitate as hydroxides in the following order: Fe, Al, Cu, Ni, Co, Li, Mn and this correlates with previous findings in literature^29,40^. Results from the NaOH precipitation at 30 and 60 °C in a solution containing 4 g/L of Al are displayed in Fig. 1 an indicate that at 30 °C iron was completely precipitated at pH 4.5, whereas at 60 °C this precipitation occurred already at pH 4, as suggested previously by Wang et al.^29^. Conversely, in the case of other metals, the temperature was found to have a less significant effect on their precipitation pH. The precipitation yield increased with temperature also at all pH values for nickel and cobalt due to more rapid reaction kinetics, while lithium and manganese precipitation yield remained similar both at 30 and 60 °C.

**Figure 2:**
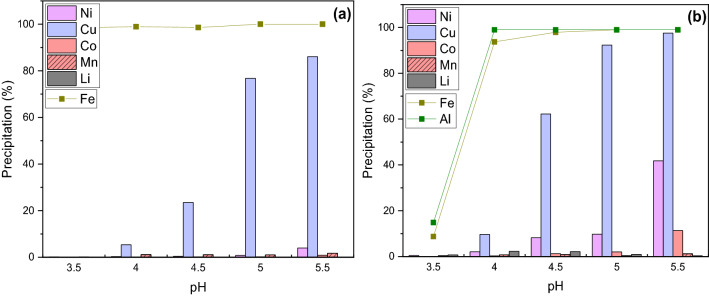
Precipitation using NaOH at 60 °C with (**a**) 0 and (**b**) 2 g/L of Al in the solution.

The effect of aluminum in the solution (0, 2 and 4 g/L) was investigated and the results are presented in Fig. 1b and Fig. 2 (T = 30 °C and 60 °C). It can be seen that 98% of iron was already precipitated in the absence of aluminum (Fig. 2A) at pH < 3.5, whereas in the presence of aluminum, iron was only precipitated completely at pH 4.5 (Figs. 1b, 2b). Bazilevskaya et al.^41^ found in their study that at initial 0–4 mol% ratio of Al^3+^ to Fe^3+^, Fe^3+^ hydrolyzed at lower pH compared to Al^3+^, and that the pH equilibrium was achieved at ~ 2.5. In contrast, when a 100 mol% ratio of Al^3+^ to Fe^3+^ was used, the resultant equilibrium was achieved at a pH proximal to 4 and such a wide shift in the precipitation pH correlates with the findings outlined in this study. In the study conducted by Bazilevskaya et al.^41^ it was found that the amount of aluminum in relation to iron affected both the initial precipitation pH as well as the final product. In the presence of ≥ 25 mol% of aluminum in relation to iron in the solution, Al(OH)_3_ was found to form initially (analyzed by ATR-FTIR). In different scenario, when < 20 mol% of aluminum to iron was present initially, ferrihydrite was the initial precipitate, although aluminum was also found to co-precipitate with iron hydroxide (analyzed by XRD and ATR-FTIR). Moreover, the tendency of aluminum to aggregate into clusters on the surface or inside the iron phases was also confirmed^41^. In the current study, 2 g/L of Al^3+^ to 1.3 g/L of Fe^3+^ corresponds to 318 mol% of Al, suggesting that Al(OH)_3_ is hypothesized to be the dominant phase. Furthermore, decreased selectivity between Cu and Fe-Al hydroxide cake was evident in our study (Fig. 2) as more than double the amount of Cu was found to precipitate at pH 4 and 4.5 in the presence of 2 g/L of Al compared to test in the absence of dissolved aluminum. In contrast, the loss of manganese was not found to be significant (Fig. 1b) in the presence of the highest Al concentration used in this study (4 g/L), as less than 5% was coprecipitated. Additionally, nickel, cobalt, and lithium loss at pH 4 increased when aluminum was present in the solution. In the absence of aluminum, the losses were < 1% (Fig. 2a) whereas in the presence of 4 g/L of dissolved aluminum the losses were 9% of Ni^2+^, 4% of Co^2+^ and 10% of Li^+^ (Fig. 1b).

**Figure 3:**
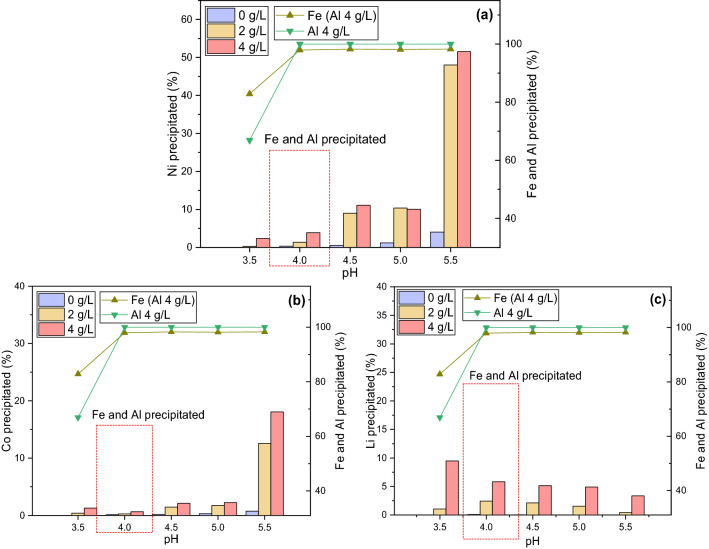
Precipitation of (**a**) Ni, (**b**) Co and (**c**) Li with LiOH as a function of Al concentration (0, 2, 4 g/L) in the solution.

Experiments were repeated with LiOH also utilized as an alternative precipitant (at 60 °C) to prevent the accumulation of Na^+^ in the solution and to determine the losses to the Fe-Al cake in comparison to NaOH. Results show that the precipitation of Fe and Al in the presence of dissolved aluminum occurs at pH 4, for both NaOH and LiOH (Figs. 2, 3). In contrast, the precipitation of nickel and cobalt only commenced at pH 5 in the absence of aluminum, whereas lithium was found not to undergo precipitation—a similar behavior to that observed with NaOH. With 2 g/L of aluminum, a minor loss of nickel and cobalt was found at pH < 4 and with 4 g/L of aluminum, Fe-Al cake was found to start to already precipitate at pH 3.5 (Fig. 3), which is similar to that of NaOH (Figs. 1, 2). It can be concluded that in the presence of dissolved aluminum the loss of nickel and lithium into the Fe-Al cake is quite significant (4% and 6%) at pH 4 and does not allow good separation between less valuable metals (Fe and Al) and the valuable metals of Ni, Co, and Li.

The results with both NaOH and LiOH show that the loss of Li^+^ into the Al(OH)_3_ cake is highest at pH 3.5 and 4, (Figs. 1, 3c), suggesting that there is potential for substantial losses during iron and aluminum precipitation, although the incorporation of Li^+^ significantly decreases with the increase in pH. A similar phenomenon has been previously observed by Hamzaoui et al.^42^ in their study on lithium recovery from natural brines using aluminum hydroxide. For example, at pH 4.3, 30% of Li^+^ was found to be absorbed by aluminum hydroxide and lithium content in the cake residue decreased with increasing pH until 5.5 when lithium was fully released to the solution. Once pH 7.4 was reached, over 90% of lithium was found to reabsorb to the aluminum compounds. The loss of Li at pH 4 can be attributed to the fact that fresh amorphous aluminum hydroxide is an effective sorbent for Li^+^ ions as the small Li^+^ is able to intercalate into the hydroxide compound^43,44^. The further increase in pH can promote the thickening and crystallization of gel, which decreases its sorptive properties resulting in deintercalation and release of lithium ions from the aluminum hydroxide^43^. In addition, the filtration of the cakes obtained at all pH values was significantly more challenging in the presence of aluminum as filter paper with the larger pore size (22 μm) was required due to the formation of aluminum hydroxide gel-like colloids^39,43^. Conversely, for the filtration of precipitated iron only, solids passed through the initially selected filter paper, therefore paper with a reduced pore size of 2.7 μm was used and the filtration was still significantly faster than any aluminum-containing slurry. This could be attributed to the formation of crystalline iron oxide, such as goethite^45^, which is known to possess a defined crystal structure that improves filtration properties.

## Discussion

Large fractions of LIB black mass (e.g., over 1 mm) are not always discarded due to the presence of copper and aluminum that carries valuable metal oxide coated on its surface^28^. For example, in a previous study by Chernyaev et al.^46^, a coarse fraction of black mass (1.250 mm) was shown to have considerably higher extraction of Co, Ni and Mn due to the presence of self-reductive elements such as Cu, Fe and Al^19^. These experimental results clearly highlight the issues related to the hydroxide co-precipitation of battery metals as a function of aluminum present in the PLS. In recycling process this phenomenon increases losses of battery metals out from the material circulation. It was shown that aluminum concentration in the PLS affects the precipitation behavior of iron, lithium, nickel, cobalt, and manganese. Moreover, the presence of aluminum causes colloidal precipitates that result in extremely slow filtration. This can challenge a potential upscaling of the solution purification unit process. In the absence of aluminum, iron could be precipitated with hydroxide efficiently and removed by filtration, whereas the presence of aluminum (≥ 2 g/L) was associated with valuable metal losses along co-precipitation.

**Figure 4:**
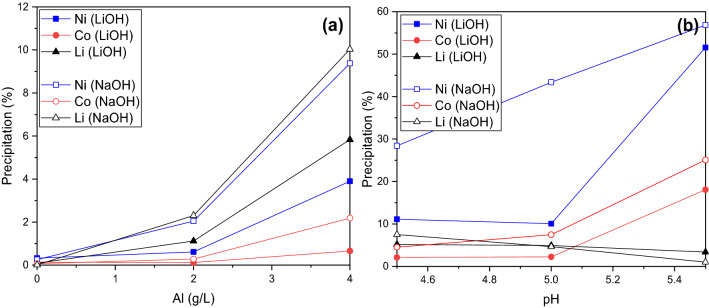
Precipitation of Ni, Co and Li (**a**) as a function of Al concentration (0, 2, 4 g/L) in the solution (pH 4) and (**b**) as a function of pH (4 g/L of Al) at 60 °C.

Figure 4 presents the precipitated amount (%) of Ni, Co, and Li the occurs at pH 4 when either NaOH or LiOH are used as precipitants. The results demonstrate that NaOH results in ca. 50% higher loss of valuable metals (Ni, Co, Li) in Fe-Al hydroxide cake, when compared to LiOH at both 2 and 4 g/L of Al. According to Fig. 4a, 2 g/L of Al in the solution causes up to 2% of metal loss to the hydroxide cake, whereas with a concentration of 4 g/L Al there is an up to 10% loss of the valuable metals.

The results detailed here highlights the importance of reducing final concentration of aluminum in the PLS to < 2 g/L, to minimize the impact of co-precipitation and filtration issues. This can be addressed by LIB waste pretreatment such as screening and/or use of lower solid concentration in leaching. Alternatively, new solution purification methods need to be developed to mitigate the challenges related to aluminum hydroxide, e.g., use of solvent extraction or alternative precipitating agents to hydroxides. Additionally, the importance of internal components separation should not be neglected. For example, Po et al.^47^ and Marshall et al.^48^ disassembled battery cells and demonstrated that all parts of the cell can be reclaimed at a high purity for further processing. However, the separation of anode and cathode from current collectors is labor intensive, therefore the battery cells need to be redesigned to improve the ease of disassembly and aid the delamination process. These issues can be overcome by ultrasound-assisted delamination processes, which can potentially handle large volumes of batteries. A new method developed by Lei et al.^49^ uses high intensity ultrasonication to separate graphite and the active material from current collector foils. This pre-treatment method results in high-purity fractions of the active material and graphite, with Al and Cu separated prior to metallurgical treatment. Liivand et al.^18^ in their research found that graphite separated from copper foil has a high potential to be recycled and used as a catalyst for electrochemical oxygen reduction reaction. Thompson et al.^15^ in their study found that such pre-treatment processes that aim to recover the active material and current collectors had lower operational costs, with 20–85% vs. 2–19% cost of recycling saved for disassembled and shredded batteries, respectively. High product purity is achieved through the suppression of current collector dissolution and lixiviant reuse.

## Conclusions

Investigation of synthetic waste battery leach solution purification revealed a strong correlation between increased aluminum concentration in solution and the co-precipitation of copper, nickel, cobalt and lithium at pH 4. At this pH iron and aluminum were completely precipitated, but also co-precipitation of battery metals is possible, compromising good battery metal extractions. In the absence of aluminum, iron was found to already precipitate at pH 3.5. However, in the presence of 2–4 g/L of aluminum in PLS at pH 4, nickel, cobalt and lithium loss to the cake were 2–10%, 1–2% and Li 2–10%. These findings demonstrate that the hydroxide precipitation of iron and aluminum is not selective towards Li^+^, Cu^2+^ and Ni^2+^ in the presence of aluminum (> 2 g/L). Moreover, the absence of aluminum in the PLS would also improve the selectivity of iron separation and its filtration efficiency. In terms of precipitant, LiOH is suggested to result in lower co-precipitation, and furthermore the accumulation of Na^+^ can be avoided. To avoid such issues caused by impurities, as well as reduce the solution purification steps and hence costs, it is of utmost importance to develop legislation, redesign and standardize battery structure and chemistry. Such steps would support the introduction of delamination and separation technologies on an industrial scale to enable the efficient recovery of all the valuable elements and battery components.
